# Recombinant Humanized IgG1 Antibody Protects against oxLDL-Induced Oxidative Stress and Apoptosis in Human Monocyte/Macrophage THP-1 Cells by Upregulation of MSRA via Sirt1-FOXO1 Axis

**DOI:** 10.3390/ijms231911718

**Published:** 2022-10-03

**Authors:** Qi Zhang, Zhonghao Li, Xianyan Liu, Ming Zhao

**Affiliations:** 1 The First School of Clinical Medicine, Southern Medical University, Guangzhou 510515, China; 2 Department of Pathophysiology, Key Lab for Shock and Microcirculation Research of Guangdong, School of Basic Medical Sciences, Southern Medical University, Guangzhou 510515, China

**Keywords:** oxidative stress, apoptosis, MSRA, SIRT1-FOXO1 axis

## Abstract

Oxidized low-density lipoprotein (oxLDL)-induced oxidative stress and apoptosis are considered as critical contributors to cardiovascular diseases. Methionine sulfoxide reductase A (MSRA) is a potent intracellular oxidoreductase and serves as an essential factor that protects cells against oxidative damage. Here, we firstly provide evidence that recombinant humanized IgG1 antibody treatment upregulated the expression of MSRA in THP-1 cells to defend against oxLDL-induced oxidative stress and apoptosis. It was also observed that the upregulation of MSRA is regulated by the forkhead box O transcription factor (FOXO1), and the acetylation of FOXO1 increased when exposed to oxLDL but declined when treated with recombinant humanized IgG1 antibody. In addition, we identified that silent information regulator 1 (SIRT1) suppresses FOXO1 acetylation. Importantly, SIRT1 or FOXO1 deficiency impaired the anti-oxidative stress and anti-apoptotic effect of recombinant humanized IgG1 antibody. Together, our results suggest that recombinant humanized IgG1 antibody exerts its anti-oxidative stress and anti-apoptotic function by upregulation of MSRA via the Sirt1-FOXO1 axis.

## 1. Introduction

Atherosclerosis has been the leading cause of cardio-cerebrovascular disease with increasing morbidity and mortality [1,2]. Atherosclerosis is a complex disease that involves chronic inflammation in combined with metabolic risk factors and autoimmune response [3]. Atherosclerosis is characterized by activated monocytes/macrophages ingesting oxidized lipids, such as oxidized low-density lipoprotein (oxLDL), and becoming lipid-laden “foam” cells; this results in the production of proinflammatory cytokines and chemokines, further leading to progressive necrotic lipid core formation and eventually developing into fibrous plaque. Moreover, macrophages decrease phagocytic activity and undergo polarization to pro-inflammation macrophages upon oxLDL recognition and internalization [4]. Apoptosis of lesional monocytes/macrophages and their defective function on cleaning up dead cells leads to plaque instability and thrombosis [5]. Since monocytes/macrophages play a central role in the stability of the atherosclerotic plaque, it is necessary to clarify the underlying apoptosis mechanism of the lesional monocytes/macrophages. Moreover, it is known that mitochondrial DNA mutations also contribute to atherosclerosis initiation and progression [6].

Proteins with sulfur-containing amino acids cysteine and methionine residues are particularly sensitive to oxidation by reactive oxygen species (ROS) and this oxidation process can subsequently be reduced by the action of certain reductases [7]. Particularly, oxidation of methionine results in two enantiomers [8], namely S-form or R-form of oxidized methionine sulfoxide. Methionine sulfoxide reductase A (MSRA) is one of methionine sulfoxide reductases that are responsible for reducing methionine sulfoxide to methionine in proteins [9]. MSRA specifically reduces the S-form of both free and peptide-bound methionine sulfoxide. Interestingly, the essential role of MSRA in defending against oxidative stress has been studied extensively. It has been reported that MSRA could decrease susceptibility to oxidative stress and increase longevity in Drosophila [10,11], yeast [12], and mice [13,14]. However, the detailed mechanism remains unknown.

The forkhead box O (FOXO) transcription factors in mammals comprise four members, namely FOXO1, FOXO3, FOXO4 and FOXO6, which are involved in regulating many cells’ physiological processes, such as oxidative stress, apoptosis, cell cycle, and cell survival and differentiation [15]. FOXO1 is mainly expressed in insulin-responsive tissues, such as pancreas, liver, skeletal muscle and adipose tissue, and also acts as a master regulator of energy metabolism [16]. As a transcription factor, the activity of FOXO1 is primarily dependent on its protein post-translational modifications, such as phosphorylation and acetylation. It has been reported that silent information regulator 1 (SIRT1), a nicotinamide adenine dinucleotide (NAD+)-dependent histone deacetylase, deacetylates FOXO1 and regulates its activity [17,18]. Therefore, it may be a promising strategy to attenuate oxidative stress and apoptosis through improving the SIRT1-FOXO1 axis.

We previously had reported that the recombinant humanized IgG1 antibody reduces atherosclerosis in ApoE^−/−^ mice fed with a high-fat-diet after immune therapies, and activates macrophage polarization from M1 toward M2 [19]. The purpose of the present work was to determine the modulation of expression of MSRA upon exposure of THP-1 cells to oxLDL. We found that recombinant humanized IgG1 antibody alleviated oxLDL-mediated THP-1 cells’ apoptosis by upregulation of MSRA. We also demonstrated that recombinant humanized IgG1 antibody decreased FOXO1 acetylation by targeting SIRT1 expression. Correspondingly, ectopic SIRT1 expression blocked oxLDL-induced FOXO1 acetylation and THP-1 cells’ apoptosis. Our data highlight a functional role of recombinant humanized IgG1 antibody in protection against oxidative stress and apoptosis and uncover its possible mechanisms.

## 2. Results

### 2.1. Recombinant Humanized IgG1 Antibody Alleviates oxLDL-Induced Oxidative Stress and Apoptosis in THP-1 Cells

**Figure 1:**
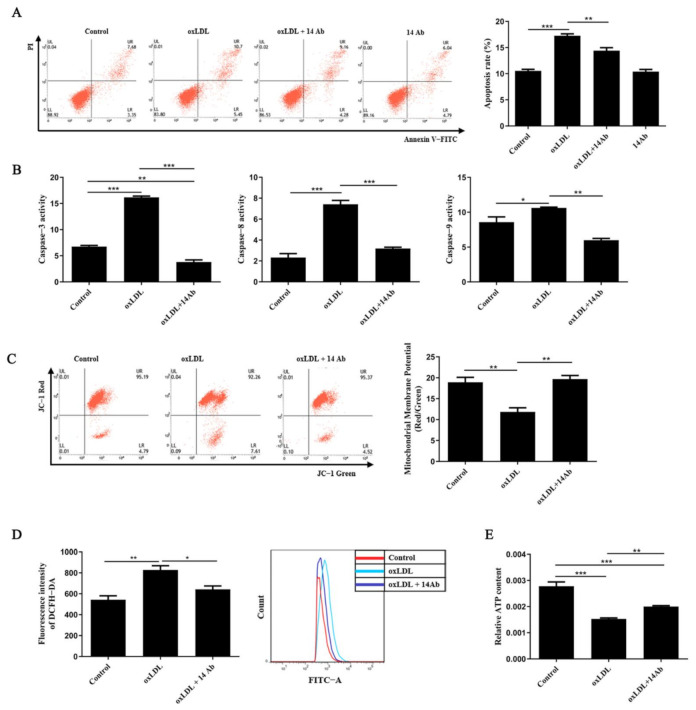
Recombinant humanized IgG1 antibody alleviates oxLDL-induced oxidative stress and apoptosis. THP-1 cells were treated with oxLDL (50 µg/mL) along with or without the recombinant humanized IgG1 antibody (14 Ab, 100 µg/mL), or just treated with recombinant humanized IgG1 antibody (14 Ab, 100 µg/mL) for 8 h, and then the cells were collected to determine apoptosis (**A**), the activity of caspase-3/8/9 (**B**), mitochondrial membrane potential (**C**), ROS production (**D**) and ATP content (**E**), respectively. Data are shown as mean ± SD (n = 4). *, *p* < 0.05; **, *p* < 0.01; ***, *p* < 0.001. oxLDL, oxidized low-density lipoprotein; ROS, reactive oxygen species.

Our previous work has revealed that recombinant humanized IgG1 antibody inhibits oxLDL-induced apoptosis in CD14^+^ monocytes derived from human peripheral blood (data not shown). To determine whether the antibody has the same effect on human monocyte/macrophage cell line THP-1, we treated the THP-1 cells with oxLDL along with or without recombinant humanized IgG1 antibody (14 Ab) to detect apoptosis by flow cytometry for Annexin V staining. As shown in Figure 1A, exposure to oxLDL led to a significant increase in apoptosis induction (16.15%) compared to normal control group (11.03%); 14 Ab intervention moderately but statistically significantly alleviated oxLDL-induced apoptosis (13.44%). Treatment of THP-1 cells with just 14 Ab did not have the anti-apoptotic effect in comparison with the control experiment (Figure 1A). In addition, oxLDL treatment enhanced caspase-3 activation, which was reversed by 14 Ab. A similar result was observed regarding the activation of caspase-8 and caspase-9, the two major upstream initiators of caspase-3 in the extrinsic and intrinsic pathway of apoptosis, respectively (Figure 1B). Moreover, 14 Ab treatment restored the mitochondrial transmembrane potential, inhibited ROS production (characterized by the fluorescence intensity of the DCFH-DA), and enhanced ATP content (Figure 1C-E). These results suggest that recombinant humanized IgG1 antibody not only exerts an anti-apoptosis effect on oxLDL-induced apoptosis, but also helps cells to resist oxidative stress in presence of oxLDL.

### 2.2. OxLDL Reduces MSRA Protein Expression in THP-1 Cells, While Recombinant Humanized IgG1 Antibody Upregulates MSRA Expression

**Figure 2:**
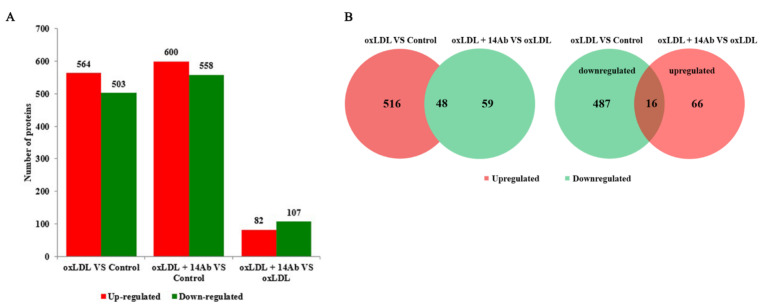
oxLDL reduces MSRA protein expression in THP-1 cells, while recombinant humanized IgG1 antibody upregulates MSRA expression. CD14^+^ monocytes were treated with oxLDL (50 µg/mL) with or without the recombinant humanized IgG1 antibody (14 Ab, 100 µg/mL) for 48 h and subject to TMT-labeled quantitative proteomics analysis. Only when the variation of proteins’ abundance was more than 1.2 times and *t*-test *p*-value < 0.05, were they accepted as differentially expressed proteins. (**A**,**B**) The number of upregulated and downregulated proteins in pairwise comparison (n = 3). oxLDL, oxidized low-density lipoprotein; TMT, Tandem Mass Tags.

**Figure 3:**
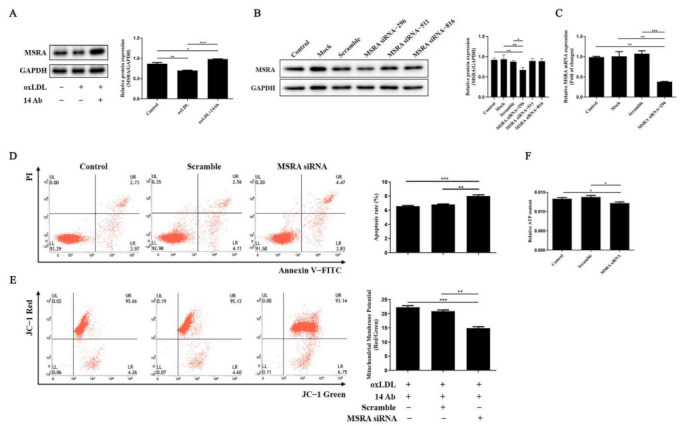
Recombinant humanized IgG1 antibody alleviates oxLDL-induced oxidative stress and apoptosis is MSRA dependent. (**A**) THP-1 cells were treated with oxLDL (50 µg/mL) with or without the recombinant humanized IgG1 antibody (14 Ab, 100 µg/mL) for 48 h, and then the cells were collected to determine the protein expression of MSRA; GAPDH was used as internal control (n = 4). (**B**,**C**) THP-1 cells were transfected with scramble or MSRA siRNA for 48 h to identify whether endogenous MSRA protein expression and mRNA transcription was blocked, respectively (n = 3). (**D**–**F**) THP-1 cells were transfected with scramble or MSRA siRNA for 48 h, following subjection to recombinant humanized IgG1 antibody (14 Ab, 100 µg/mL) along with oxLDL (50 µg/mL) for 8 h to check cells apoptosis, mitochondrial membrane potential and ATP content, respectively (n=4). Data are shown as mean ± SD. *, *p* < 0.05; **, *p* < 0.01; ***, *p* < 0.001. oxLDL, oxidized low-density lipoprotein; MSRA, methionine sulfoxide reductase A.

To investigate the underlying mechanism of recombinant humanized IgG1 antibody in anti-apoptosis and anti-oxidative stress, TMT-labeled quantitative proteomics were applied to compare the protein expression profiles between any two groups (control, oxLDL, and oxLDL + 14 Ab) in isolated and purified human peripheral blood CD14^+^ monocytes. A total of 5116 proteins were identified, of which 4428 proteins were quantitated. The property and functions of qualified proteins were classified with Gene Ontology (GO) and Cluster analysis of GO, KEGG pathway and protein domain (Supplementary Materials). Only when the variation of those proteins’ abundance was more than 1.2 times and *t*-test *p*-value < 0.05, were they accepted as differentially expressed proteins. The number of upregulated and downregulated proteins in pairwise comparison was shown in Figure 2A. Finally, we found 16 proteins that were downregulated in oxLDL treatment compared to normal control group, but upregulated in oxLDL plus 14 Ab group compared to oxLDL only group (Figure 2B). Among the 16 candidates, methionine sulfoxide reductase A (MSRA), which functions as a repair enzyme for proteins that have been inactivated by oxidation, aroused our great interest (Table 1). We further verified that 14 Ab treatment along with oxLDL in THP-1 cells significantly reversed the reduction of MSRA protein expression induced by oxLDL only, which is consistent with the proteomics sequencing results (Figure 3A).

### 2.3. Recombinant Humanized IgG1 Antibody Alleviates oxLDL-Induced Oxidative Stress and Apoptosis in THP-1 Cells Is MSRA Dependent

We then designed siRNA to block the endogenous MSRA expression to determine whether MSRA is involved in apoptosis regulation. As demonstrated in Figure 3B and C, the endogenous MSRA protein expression and mRNA transcription was knocked down when applied to MSRA siRNA-296, so we chose MSRA siRNA-296 for subsequent working conditions. As shown in Figure 3D–E, transfection with MSRA siRNA-296 increased cell apoptosis and reduced mitochondrial membrane potential. Meanwhile, the enhancement of ATP content induced by 14 Ab was potently downregulated due to the reduced expression of MSRA (Figure 3E). These results suggested that the anti-apoptosis and anti-oxidant stress properties of 14 Ab are mediated by the upregulation of MSRA when cells are incubated with oxLDL.

### 2.4. The Upregulation of MSRA Is Regulated by Transcription Factor FOXO1

**Figure 4:**
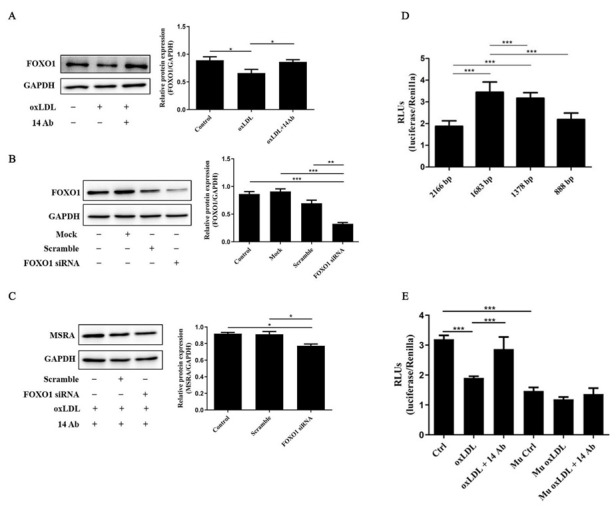
The upregulation of MSRA is regulated by transcription factor FOXO1. (**A**) THP-1 cells were treated with oxLDL (50 µg/mL) with or without the recombinant humanized IgG1 antibody (14 Ab, 100 µg/mL) for 48 h, and then the cells were collected for determine the protein expression of FOXO1; GAPDH was used as internal control (n = 6). (**B**) THP-1 cells were transfected with scrambled or FOXO1 siRNA for 48 h to identify whether endogenous FOXO1 was blocked by Western blot (n = 3). (**C**) THP-1 cells were transfected with scrambled or FOXO1 siRNA for 48 h, followed by treatment with the recombinant humanized IgG1 antibody (14 Ab, 100 µg/mL) along with oxLDL (50 µg/mL) for another 48 h to measure the expression of MSRA (n = 4). (**D**) Construction of MSRA reporter gene vectors of different length and transfection into HepG2 cells for 24 h to determine the luciferase activity (n = 6). (**E**) Mutation of FOXO1 binding site in MSRA promoter (1683bp) and transfection into HepG2 cells for 24 h, followed by treatment with oxLDL (50 µg/mL) with or without the recombinant humanized IgG1 antibody (14 Ab, 100 µg/mL) for another 24 h to measure the luciferase activity (n = 6). Data are shown as mean ± SD. *, *p* < 0.05; **, *p* < 0.01; ***, *p* < 0.001. oxLDL, oxidized low-density lipoprotein; MSRA, methionine sulfoxide reductase A; FOXO1, Forkhead box O1.

Evidence has shown that the transcription factor FOXO1 regulates a number of cellular processes, such as cell cycle progression, apoptosis and oxidative stress [15]. We therefore investigated whether FOXO1 participates in the effects of 14 Ab on MSRA expression and MSRA-mediated anti-apoptosis and anti-oxidant stress. The results showed that 14 Ab significantly enhanced the protein levels of FOXO1 in THP-1 cells (Figure 4A). Thereafter, THP-1 cells were transfected with FOXO1 siRNA for 48 h, followed by adding 14 Ab along with oxLDL. As indicated in Figure 4B and C, transfection of THP-1 cells with FOXO1 siRNA reduced the protein level of FOXO1, and almost abolished the amplification of 14 Ab on MSRA expression. Moreover, we cloned 4 different lengths of the MSRA promoter region into pGL3-basic vector to obtain the human MSRA reporter and co-transfected into the HepG2 cells together with renilla luciferase control, as THP-1 cells were difficult to transfect transiently. The results indicated that the 1683bp MSRA reporter obtained the highest luciferase activity, followed by 1378bp MSRA reporter compared with 2166bp MSRA reporter (Figure 4D). Next, we transfected the 1683bp MSRA reporter into HepG2 cells for 24 h, followed by adding oxLDL with or without 14 Ab for another 24 h, and found that 14 Ab treatment significantly restored the reduction of MSRA promoter activity induced by oxLDL (Figure 4E). However, the mutation of the FOXO1 binding site on 1683bp MSRA reporter predicted by JASPAR website almost eliminated the luciferase activity, even though in the presence of 14 Ab (Figure 4E). These results suggested that 14 Ab enhanced MSRA expression in the presence of oxLDL is regulated by the transcription factor FOXO1.

### 2.5. Recombinant Humanized IgG1 Antibody Alleviates oxLDL-Induced Apoptosis in THP-1 Cells via Modulation of SIRT1-Dependent FOXO1 Deacetylation

**Figure 5:**
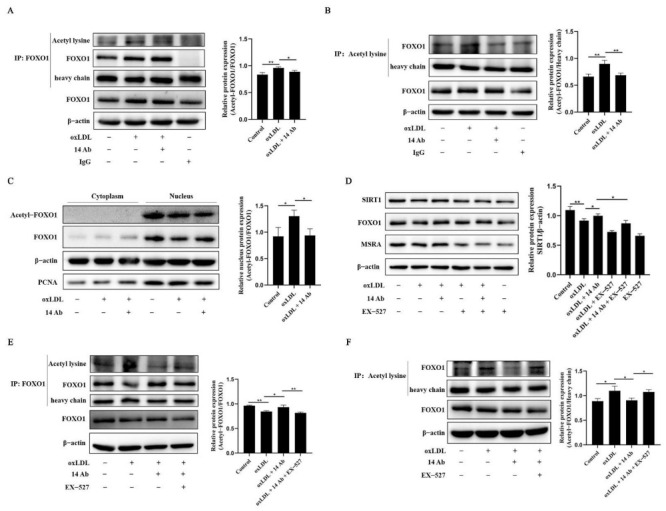

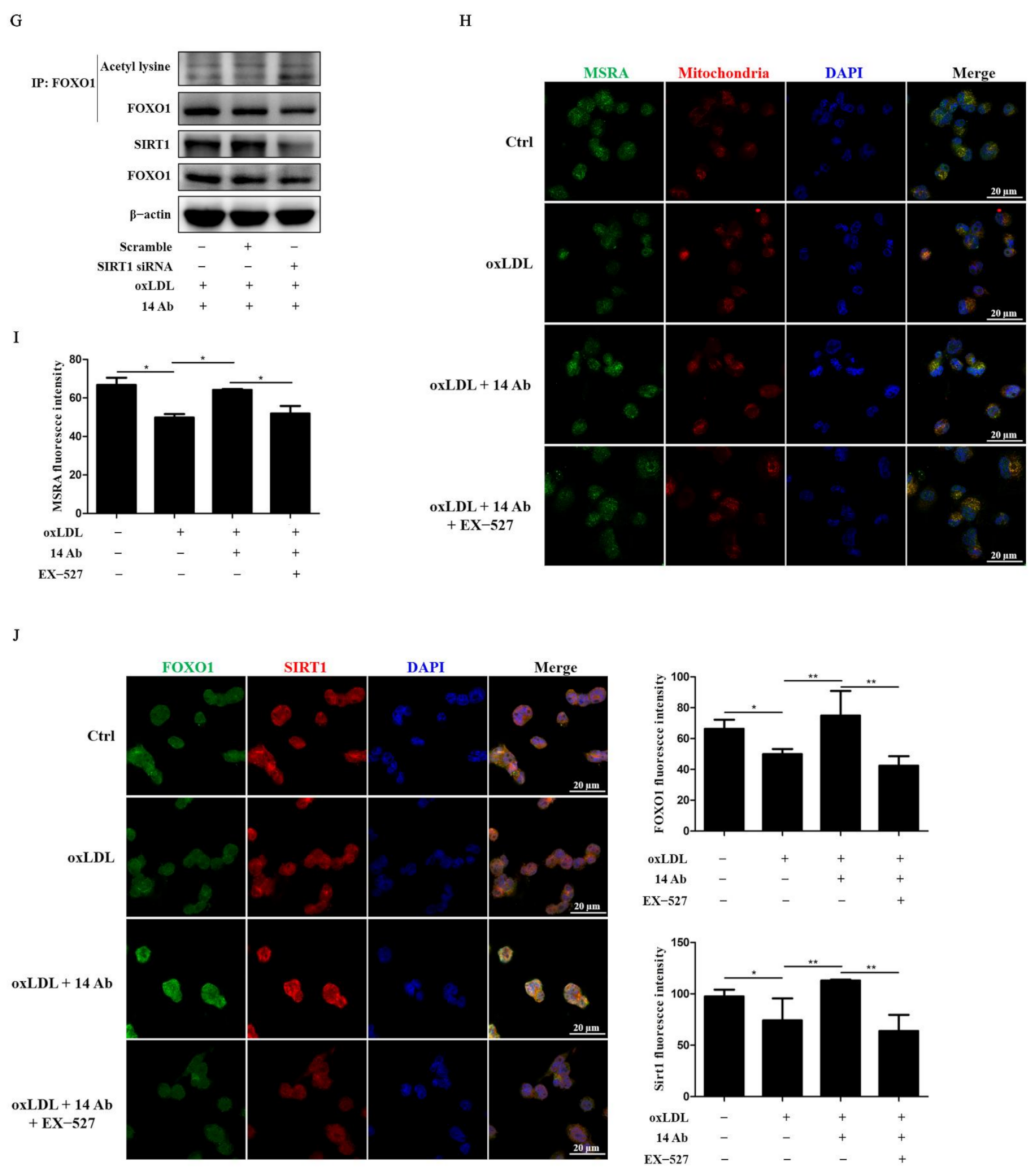
Recombinant humanized IgG1 antibody ameliorates oxLDL-induced apoptosis via modulation of SIRT1-dependent FOXO1 deacetylation. (**A**,**B**), THP-1 cells were treated with oxLDL (50 µg/mL) along with or without the recombinant humanized IgG1 antibody (14 Ab, 100 µg/mL) for 48 h, and then the cells were collected for immunoprecipitation to determine the acetylation modification of FOXO1; IgG was used as an isotype control (n = 4). (**C**) THP-1 cells were treated with oxLDL (50 µg/mL) with or without the recombinant humanized IgG1 antibody (14 Ab, 100 µg/mL) for 48 h, and then the cells were collected for separation of cytoplasmic and nuclear proteins and to determine the acetylation modification of FOXO1 as well (n = 4). (**D**) THP-1 cells were pretreated with SIRT1 inhibitor EX-527 (10 µM) for 30 min, or following treatment with oxLDL (50 µg/mL) along with or without the recombinant humanized IgG1 antibody (14 Ab, 100 µg/mL) for 48 h to measure the protein expression of SIRT1, FOXO1 and MSRA (n = 4). (**E**,**F**), THP-1 cells were pretreated with SIRT1 inhibitor EX-527 (10 µM) for 30 min, followed by treatment with oxLDL (50 µg/mL) with the recombinant humanized IgG1 antibody (14 Ab, 100 µg/mL) or treated with oxLDL alone or with the recombinant humanized IgG1 antibody (14 Ab, 100 µg/mL) for 48 h to measure the acetylation modification of FOXO1 (n = 3). (**G**) THP-1 cells were transfected with scrambled or SIRT1 siRNA for 48 h, followed by treatment with oxLDL (50 µg/mL) along with the recombinant humanized IgG1 antibody (14 Ab, 100 µg/mL) for another 48 h, and then the cells were collected for immunoprecipitation to determine the acetylation modification of FOXO1 (n = 4). (**H**) The cell treatment was as indicated in (**E**). The cells were fixed and permeabilized for immunofluorescence staining for MSRA and mitochondrial membrane potential. Images were taken using a ZESIS confocal fluorescent microscope. (**I**) The relative fluorescence intensity of (**H**) was determined by ZEN 3.4 software (ZEN 3.4 blue edition, Carl Zeiss, Oberkochen, Germany). (**J**) The cell treatment was as indicated in (**E**). The cells were fixed and permeabilized for immunofluorescence staining for FOXO1 and SIRT1 (left panel), and statistical analysis of fluorescence intensity (right panel). Data are shown as mean ± SD. *, *p* < 0.05; **, *p* < 0.01; oxLDL, oxidized low-density lipoprotein; MSRA, methionine sulfoxide reductase A; FOXO1, Forkhead box O1; SIRT1, silent information regulator 1.

**Figure 6:**
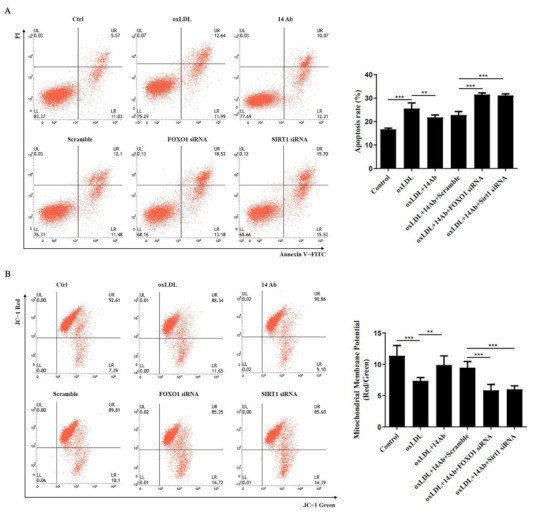
Recombinant humanized IgG1 antibody ameliorates oxLDL-induced apoptosis via SIRT1-FOXO1 axis. THP-1 cells were transfected with FOXO1 siRNA or SIRT1 siRNA for 48 h, and then subjected to oxLDL (50 µg/mL) with or without recombinant humanized IgG1 antibody (14 Ab, 100 µg/mL) for 8 h to check cells apoptosis (**A**) and mitochondrial membrane potential (**B**). Data are shown as mean ± SD (n = 3). **, *p* < 0.01; ***, *p* < 0.001. oxLDL, oxidized low-density lipoprotein; FOXO1, Forkhead box O1; SIRT1, silent information regulator 1.

Since the acetylation modification attenuates the ability of FOXO1 to bind DNA and suppress transcription [20,21], we examined the FOXO1 acetylation in oxLDL-induced apoptosis. As shown in Figure 5A and B, THP-1 cells exposed to oxLDL manifested remarkably increased FOXO1 acetylation, while treatment with recombinant humanized IgG1 antibody could reverse the acetylation modification of FOXO1 induced by oxLDL. Moreover, compared with oxLDL treatment alone, recombinant humanized IgG1 antibody treatment clearly elevated the nuclear level of FOXO1 but reduced the nuclear level of FOXO1 acetylation oxLDL (Figure 5C). Interestingly, SIRT1, a deacetylase responsible for FOXO1 deacetylation [22], was upregulated by recombinant humanized IgG1 antibody (Figure 5D). Importantly, we also found that EX-527, which blocks SIRT1 activity, decreased the expression of FOXO1 and MSRA (Figure 5D, H–J), as well as increased the level of acetylated FOXO1 (Figure 5E and F). In addition, knockdown of SIRT1 exacerbated FOXO1 acetylation (Figure 5G). These findings strongly suggest that the increased FOXO1 acetylation result from inhibition of SIRT1 is involved in oxLDL-induced decline of MSRA expression. Consistently, when there is knockdown of SIRT1 or FOXO1, oxLDL-induced cell apoptosis was further increased, even in the presence of recombinant humanized IgG1 antibody (Figure 6). Similarly, the recombinant humanized IgG1 antibody could not inhibit the decreased mitochondrial membrane potential induced by oxLDL upon knockdown of SIRT1 or FOXO1 (Figure 6). Consequently, we concluded that the upregulation of MSRA by recombinant humanized IgG1 antibody could inhibit oxLDL-induced apoptosis via a SIRT1-FOXO1 axis.

## 3. Discussion

Intracellular redox status is tightly regulated by oxidant and antioxidant systems. An imbalance between these systems causes ROS accumulation which leads to oxidative stress and inflammation. Oxidized low-density lipoprotein (oxLDL)-induced oxidative stress and apoptosis are considered as critical contributors to cardiovascular diseases such as atherosclerosis. Various studies have shown that oxLDL promotes ROS generation in endothelial cells, vascular smooth muscle cells, and macrophages. oxLDL induces pro-inflammatory responses, pro-oxidative conditions and endothelial cell apoptosis. Macrophages are considered immune sentinels and play a role in maintaining tissue homeostasis. Histological examination of human atherosclerotic plaques has confirmed that macrophage subsets are associated with plaque progression [23]. Several drugs that inhibit atherosclerosis by targeting macrophages have been reported [24]. Regulation of macrophages may thus be a therapeutic strategy for atherosclerosis [25]. Our previous study had demonstrated that an antibody may reduce atherosclerosis by activating monocyte/macrophage polarization in ApoE^−/−^ mice [19]. In this paper we have further explored the anti-apoptotic effect of the antibody on monocytes/macrophages.

MSRA, one of the antioxidant defenses in cells, is important in the maintenance of redox homeostasis and in the prevention of oxidative stress-related disease. It has been confirmed that MSRA plays a protective role against hypoxia/reoxygenation-induced cell death in cardiac myocytes [26] and neuronal cells [9]. MSRA mutations or deficiency in *E. coli* and yeast were particularly sensitive to oxidative damage [27,28]. MSRA knockout mice are highly sensitive to oxidative stress and show nerve damage and shortened lifespans [14], whereas MSRA transgenic Drosophila show significantly enhanced anti-oxidation and anti-aging characteristics [11]. Our study demonstrates that recombinant humanized IgG1 antibody inhibits ROS production in THP-1 cells caused by oxLDL as well as protects against apoptosis. The protective functions of recombinant humanized IgG1 antibody are attributed to an increased MSRA expression, indicating that the antioxidant and anti-apoptotic effects of recombinant humanized IgG1 antibody are closely associated with MSRA.

In previous studies, it was shown that the expression of MSRA is mainly regulated by transcription factor Forkhead box group O 3a (FOXO3a) [29,30,31]; this is a member of the family of Forkhead transcription factors, which controls the expression of many endogenous antioxidant-encoding genes, including manganese superoxide dismutase and catalase [32,33]. However, our results show that FOXO1, another member of the family of Forkhead transcription factors, also participates in the regulation of MSRA expression in THP-1 cells treated with recombinant humanized IgG1 antibody. As FOXO proteins are tightly regulated to determine cell survival and cell death responsive to specific environmental conditions, we also confirm that knockdown of endogenous FOXO1 exacerbates THP-1 cells apoptosis. The activity of FOXO1 is strictly regulated by modifications on its protein, which ensures that transcription of its downstream target genes is tightly responsive to environmental signals. Phosphoinositide 3-kinase/protein kinase B (PI3K/PKB) phosphorylates FOXO1 protein results in disrupted interactions between the FOXO1 protein and its target DNA and lead to the translocation of the FOXO1 protein from the nucleus to the cytoplasm, thus suppressing FOXO1-dependent transcription. C-Jun N-terminal kinase (JNK) can also phosphorylate FOXO1, which results in the import of the FOXO1 protein from the cytoplasm to the nucleus, thereby antagonizing the action of PI3K/PKB. FOXO1 protein activity is also regulated by reversible acetylation modification. Acetylation of FOXO1 attenuates the ability of FOXO1 to bind DNA and suppress transcription. Interestingly, it was also found that acetylation regulated the function of FOXO1 by influencing its sensitivity for phosphorylation. Our data show that recombinant humanized IgG1 antibody decreased acetylation of FOXO1 induced by oxLDL, which in turn increased FOXO1 transcriptional activity.

FOXO1 acetylation is well known to be regulated by SIRT1, an NAD+-dependent class III deacetylase and widely found in human tissues and cells [17,20,34]. It has been reported that SIRT1 deacetylated FOXO1 and decreased H_2_O_2_-induced granulosa cell apoptosis [35]. Curcumin alleviates oxidative stress and inhibits apoptosis in diabetic cardiomyopathy via SIRT1-FOXO1 pathways [36]. In the present study, we also found that SIRT1 deacetylated FOXO1 in THP-1 cells upon recombinant humanized IgG1 antibody treatment, and this was suppressed by SIRT1 inhibitor EX-527. Moreover, SIRT1 deficiency impaired the protective effect of recombinant humanized IgG1 antibody against apoptosis, suggesting the SIRT1-FOXO1 axis as a potential target of recombinant humanized IgG1 antibody against oxidative stress and anti-apoptosis. We know that the antibody regulates immune responses through interacting with Fc receptors [37]. There are 4 Fc receptors of IgG, namely Fcgamma-RI, -RIIa, -RIIb, and -RIII. FcgammaRIIb is the only one which has negative signal transduction with immunoreceptor tyrosine-based in-activation motif (ITIM), while the others have a ITAM (immunoreceptor tyrosine-based activation motif) with a positive signaling transduction. We previously reported that the antibody inhibits oxLDL-induced macrophage MCP-1 release through the FcgammaRIIb signal transduction pathway [38]. Thus, whether FcgammaRIIb and its downstream signal transduction participates in the regulation of MSRA expression as well as reduction of oxLDL-induced apoptosis upon 14 Ab treatment, requires further exploration.

In conclusion, our findings for the first time imply that recombinant humanized IgG1 antibody upregulates MSRA and inhibits oxLDL-induced THP-1 cells’ apoptosis as a result of increased SIRT1 expression and decreased FOXO1 acetylation. This reveals the critical role of the SIRT1-FOXO1 axis in recombinant humanized IgG1 antibody mediated upregulation of MSRA expression and anti-apoptosis.

## 4. Materials and Methods

Dulbecco’s modified Eagle’s medium (DMEM), RPMI 1640 medium, fetal bovine serum (FBS) and phosphate-buffered saline (PBS) were purchased from Invitrogen (Carlsbad, CA, USA). The TRIzol Reagent was from Takara Bio Inc. (Kusatsu, Japan). Antibody against MSRA, FOXO1 and SIRT1 was purchased from Proteintech (Wuhan, China), Cell Signaling Technology (Danvers, MA, USA) and Abcam (Cambridge, UK), respectively. Anti-acetyl Lysine antibody was from Abcam (Cambridge, UK). Inhibitor of SIRT1 (EX-527) was from MCE (Shanghai, China). Annexin V-FITC Apoptosis Detected Kit was purchased from BD Biosciences (Franklin Lakes, NJ, USA). Mitochondrial membrane potential assay kit and ATP Assay Kit was from Beyotime (Beijing, China). DCFH-DA were purchased from Invitrogen (CA, USA). The specific siRNA for MSRA, FOXO1 and SIRT1 were designed and synthesized by GenePharma (Suzhou, China). OxLDL was purchased from Yiyuan (Guangzhou, China). The recombinant humanized IgG1 antibody (clone: NO. 14, namely 14 Ab) was produced and maintained by our laboratory, and the detailed properties of the antibody were determined in our previous study [19]. THP-1 cells were maintained in our lab.

### 4.1. Preparation of CD14^+^ Human Monocytes

Human peripheral blood mononuclear cells (PBMCs) were heparinized and layered over Ficoll-Isopaque (Pharmacia, Freiburg, Germany) density gradient reagent according to the manufacturer’s instructions. Mononuclear cells were separated by centrifugation at 400 *g* for 30 min at room temperature. Mononuclear cells were collected and washed two times with PBS without Ca^2+^ and Mg^2+^ by centrifugation at 250 g for 20 min at 4 °C. Cells were then diluted with complete RPMI 1640 medium. Human monocytes were purified with MACS CD14 microbeads (Miltenyi Biotec, Bergisch Gladbach, Germany) according to the manufacturer’s instructions.

### 4.2. Cell Culture

CD14^+^ monocytes and THP-1 cells (human monocytic leukemia cell line) were grown in RPMI 1640 medium supplemented with 10% FBS under standard culture conditions (5% CO_2_, 37 °C), and exposed to oxLDL (50 µg/mL) [39] with or without recombinant humanized IgG1 antibody (100 µg/mL) [19] for 48 h. The THP-1 cells were then collected for extraction of proteins to analyze MSRA and FOXO1 expression, while CD14^+^ monocytes were collected for proteomics analysis.

### 4.3. Protein Extraction, Digestion and Analysis

Cells were sonicated three times on ice using a high intensity ultrasonic processor (Scientz, Ningbo, China) in lysis buffer (8 M urea, 1% Protease Inhibitor Cocktail). The remaining debris was removed by centrifugation at 12,000 g at 4 °C for 10 min. Finally, the supernatant was collected and the protein concentration was determined with BCA kit according to the manufacturer’s instructions.

For digestion, the protein solution was reduced with 5 mM dithiothreitol for 30 min at 56 °C and alkylated with 11 mM iodoacetamide for 15 min at room temperature in darkness. The protein sample was then diluted by adding 100 mM TEAB to urea concentration less than 2 M. Finally, trypsin was added at 1:50 trypsin-to-protein mass ratio for the first digestion overnight and 1:100 trypsin-to-protein mass ratio for a second 4 h-digestion.

After trypsin digestion, peptide was desalted by Strata X C18 SPE column (Phenomenex, Torrance, CA, USA) and vacuum-dried. Peptide was reconstituted in 0.5 M TEAB and processed according to the manufacturer’s protocol for TMT kit. Briefly, one unit of TMT reagent was thawed and reconstituted in acetonitrile. The peptide mixtures were then incubated for 2 h at room temperature and pooled, desalted and dried by vacuum centrifugation. The tryptic peptides were fractionated into fractions by high pH reverse-phase HPLC using Thermo Betasil C18 column (5 μm particles, 10 mm ID, 250 mm length). Briefly, peptides were first separated with a gradient of 8% to 32% acetonitrile (pH 9.0) over 60 min into 60 fractions. Then, the peptides were combined into 18 fractions and dried by vacuum centrifuging.

The tryptic peptides were dissolved in 0.1% formic acid (solvent A), directly loaded onto a home-made reversed-phase analytical column (15-cm length, 75 μm i.d.). The gradient was increased from 6% to 23% solvent B (0.1% formic acid in 98% acetonitrile) over 26 min, 23% to 35% in 8 min, climbing to 80% in 3 min, then holding at 80% for the last 3 min, all at a constant flow rate of 400 nL/min on an EASY-nLC 1000 UPLC system. The peptides were subjected to NSI source followed by tandem mass spectrometry (MS/MS) in Q ExactiveTM Plus (Thermo, Waltham, MA, USA) coupled online to the UPLC.

### 4.4. Bioinformatics Analysis and Functional Enrichment

The Gene Ontology (GO) annotation proteome, containing cellular component, molecular function and biological processes, was derived from the UniProt-GOA database (www.http://www.ebi.ac.uk/GOA/, accessed on 10 January 2022). Identified proteins domain functional descriptions were annotated by InterProScan (a sequence analysis application) based on protein sequence alignment method, and the InterPro domain.

Proteins were classified by GO annotation into three categories: biological process, cellular compartment and molecular function. For each category, a two-tailed Fisher’s exact test was employed to test the enrichment of the differentially expressed protein against all identified proteins. The GO with a corrected *p*-value < 0.05 is considered significant.

The Encyclopedia of Genes and Genomes (KEGG) database was used to identify enriched pathways by a two-tailed Fisher’s exact test, to test the enrichment of the differentially expressed proteins against all identified proteins.

### 4.5. Western Blot Analysis

Western blot analysis was conducted as described previously [40]. Briefly, the total proteins were extracted, after which their concentrations were determined using a BCA kit (Beyotime; Beijing, China). The proteins (20 μg per lane) were subsequently separated by 10% SDS-PAGE and transferred to a PVDF membrane, which was immuno-blotted using antibodies against *GAPDH*, *MSRA*, *FOXO1* and *SIRT1*, respectively. Following a series of rinses in TBS-T, the membrane was incubated with a peroxidase-conjugated secondary antibody. Lastly, the proteins were visualized by enhanced chemiluminescence (ECL; Merck Millipore, Darmstadt, Germany), and the relative expression levels were assessed by Image J via densitometry.

### 4.6. Cell Transfection

THP-1 cells that had reached approximately 80% confluence were transfected with small-interfering RNA (siRNA) specific for *SIRT1, FOXO1* and *MSRA*, respectively, using Lipofectamine^TM^ 3000 (Invitrogen), according to the instructions of manufacturers. After transfection for 48 h, cells were harvested for RT-qPCR and Western blot analysis to evaluate the silencing efficiency or after treatment with recombinant humanized IgG1 antibody (100 µg/mL) with or without oxLDL (50 µg/mL) for another 24 h to detect apoptosis and mitochondrial membrane potential by FACS.

### 4.7. Luciferase Reporter Gene Assays

A 2166 bp fragment of human *MSRA* promoter was amplified by PCR from the genome of the THP-1 cells and then cloned into the reporter vector pGL3-basic (Promega, Madison, WI, USA); truncated *MSRA*-Luc vector and mutated *MSRA*-Luc vector were constructed by the same strategy. Transfection experiments were carried out in 24-well plates using Lipofectamine^TM^ 3000 (Invitrogen). 24 h after transfection, HepG2 cells were treated with or without recombinant humanized IgG1 antibody for another 24 h. A Dual-Luciferase Reporter Assay System (Promega) was used to evaluate luciferase activity, in accordance with the manufacturer’s instructions. The data was expressed as relative luciferase activity (firefly luciferase activity/renilla luciferase activity).

### 4.8. Statistical Analysis

All data were obtained from at least three indepent experiments and were analyzed via one-way ANOVA and the Student–Newman–Keuls (SNK) post hoc multiple comparison test by GraphPad Prism 6 software (GraphpadPrism6, GraphPad Software, San Diego, CA, USA). The results are expressed as the mean ± standard deviation (SD). Results with *p* < 0.05 were considered statistically significant. *, *p* < 0.05; **, *p* < 0.01; ***, *p* < 0.001.

## Figures and Tables

**Table 1:** 16 candidates were screened and exhibited after the intersectional clustering of differentially expressed protein.

Protein Accession	Protein Description	oxLDL vs. Ctrl	oxLDL + 14 Ab vs. oxLDL	oxLDL + 14 Ab vs. Ctrl
P14222	Perforin-1	0.819	1.209	0.991
Q92890	Ubiquitin recognition factor in ER-associated degradation protein 1	0.77	1.284	0.988
Q9Y385	Ubiquitin-conjugating enzyme E2 J1	0.785	1.331	1.044
Q8NCW5	NAD(P)H-hydrate epimerase	0.653	1.442	0.941
P01034	Cystatin-C	0.601	1.255	0.754
P17050	Alpha-N-acetylgalactosaminidase	0.811	1.237	1.003
P22694	cAMP-dependent protein kinase catalytic subunit beta	0.548	1.28	0.701
Q9GIY3	HLA class II histocompatibility antigen, DRB1-14 beta chain	0.674	1.281	0.864
Q13409	Cytoplasmic dynein 1 intermediate chain 2	0.818	1.307	1.07
Q9Y2E5	Epididymis-specific alpha-mannosidase	0.768	1.231	0.945
O75822	Eukaryotic translation initiation factor 3 subunit J	0.715	1.202	0.859
P28161	Glutathione S-transferase Mu 2	0.66	1.215	0.803
Q9GZP4	PITH domain-containing protein 1	0.774	1.231	0.953
Q9BUE0	Mediator of RNA polymerase II transcription subunit 18	0.649	1.307	0.848
Q9UJ68	Mitochondrial peptide methionine sulfoxide reductase	0.625	1.274	0.796
Q15661	Tryptase alpha/beta-1	0.826	1.678	1.386

## Data Availability

The data obtained in this study are available in the article.

## Supplementary Materials

The following supporting information can be downloaded at: https://www.mdpi.com/article/10.3390/ijms231911718/s1.

## Abbreviations

oxLDL: oxidized low-density lipoprotein; MSRA: methionine sulfoxide reductase A; ROS: reactive oxygen species; FOXO1: Forkhead box O1; SIRT1: silent information regulator 1; PBMCs: peripheral blood mononuclear cells.
