# Supplementary material for: Recombinant Humanized IgG1 Antibody Protects against oxLDL-Induced Oxidative Stress and Apoptosis in Human Monocyte/Macrophage THP-1 Cells by Upregulation of MSRA via Sirt1-FOXO1 Axis
Source: Int J Mol Sci. 2022 Oct 3;23(19):11718. doi: 10.3390/ijms231911718 (PMC9569918; doi:10.3390/ijms231911718)
Supplement: Supplementary file 1 [file ijms-23-11718-s001.zip › ijms-1889773-supplementary.pdf]

Supplementary Figure.

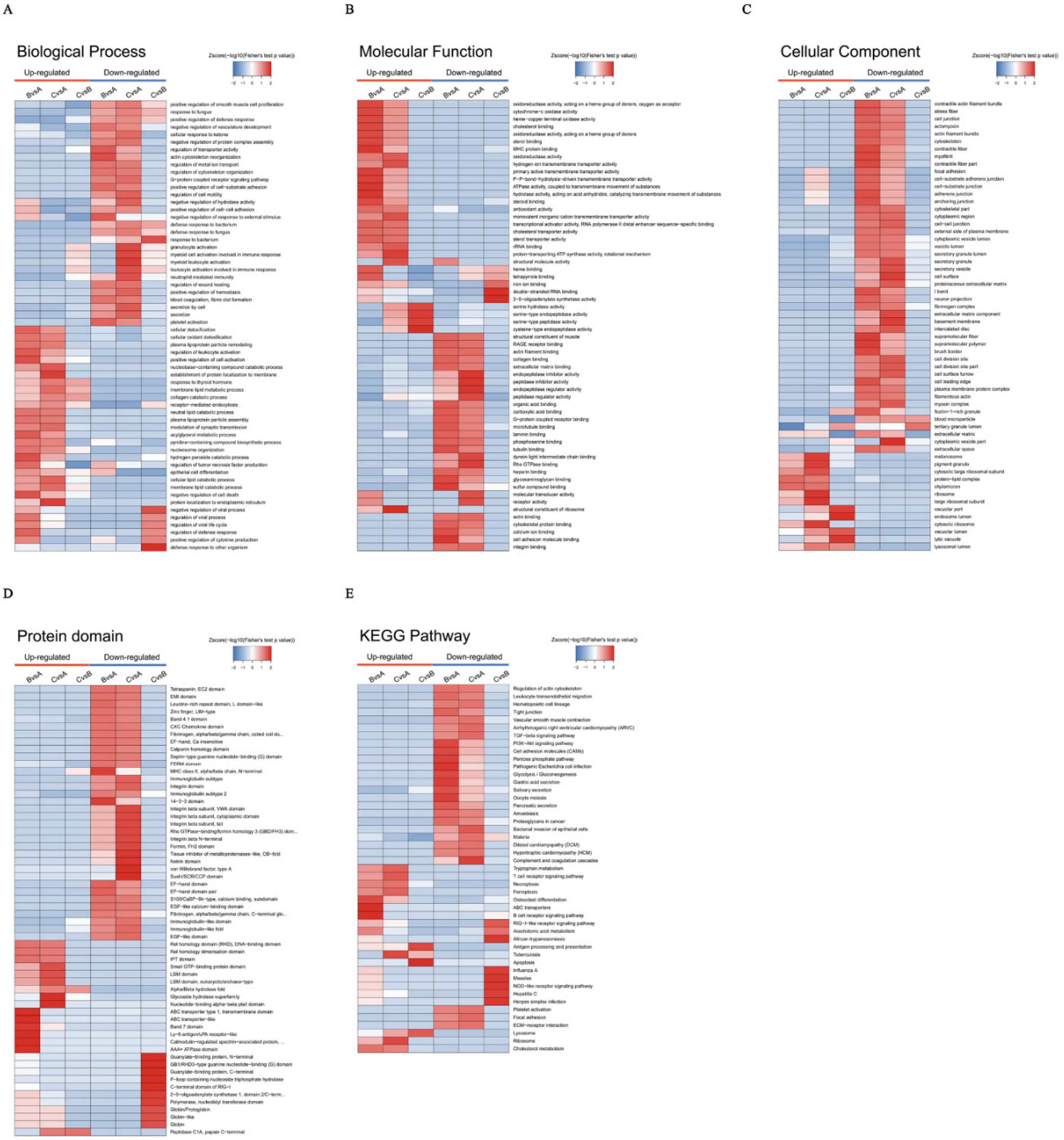

Figure S1. Functional classification of differentially expressed proteins identified by TMT-labeled quantitative proteomics analysis. The property and functioms of differentially expressed proteins proteins were classified with biological process (A), molecular function (B), cellular component (C), protein domain (D) and KEGG pathway (E); Group A-C indicates treatment CD14+ monocytes with control, oxLDL, oxLDL plus 14 Ab, respectively.
